# Genetic diversity of *Plasmodium vivax* in immigrant patients exhibiting severe and non-severe clinical manifestations in northern suburbs of Paris

**DOI:** 10.1017/S0950268823001632

**Published:** 2024-10-02

**Authors:** Adama Soumaoro, Omar Hamarsheh, Anthony Marteau, Sophie Brun, Olivier Bouchaud, Yves Cohen, Frédéric Adnet, Marc Thellier, Sandrine Houze, Arezki Izri, Mohammad Akhoundi

**Affiliations:** 1 Parasitology-Mycology Department, Avicenne Hospital, AP-HP, Bobigny, France; 2 Department of Biological Sciences, Al-Quds University, Jerusalem, Palestine; 3 Infectious diseases Department, Avicenne Hospital, AP-HP, Bobigny, France; 4 Service de Réanimation Médico-Chirurgicale, Hôpital Avicenne, Assistance Publique-Hôpitaux de Paris, Bobigny, France; 5 AP-HP, Service des Urgences et Service d’Aide Médicale Urgente, Centre Hospitalier Universitaire Avicenne, Bobigny Cedex, Francia, Université Paris 13, Sorbonne Paris Cité, Bobigny, Francia, France; 6 Sorbonne Université, INSERM, Institut Pierre-Louis d’Epidémiologie et de Santé Publique, AP-HP, Laboratoire de Parasitologie, Mycologie, Hôpital Pitié-Salpêtrière, Paris, France; 7 Service de Parasitologie-mycologie CNR du Paludisme, AP-HP Hôpital Bichat, Paris, France; 8 Unité des Virus Émergents (UVE: Aix-Marseille Univ-IRD 190-Inserm 1207-IHU Méditerranée Infection), Marseille, France

**Keywords:** genetic diversity, parasitological diagnosis, *Plasmodium vivax*, phylogenetic analysis, severe malaria complication

## Abstract

*Plasmodium vivax* is the most frequent and widely distributed cause of recurring malaria. It is a public health issue that mostly occurs in Southeast Asia, followed by the Middle East, Latin, and South Americas and sub-Saharan Africa. Although it is commonly known as an etiologic agent of malaria with mild clinical manifestations, it can lead to severe complications. It has been neglected and understudied for a long time, due to its low mortality, culturing infeasibility, and mild clinical manifestations in comparison to *P. falciparum.* Despite the mild clinical issues commonly rose for *P. vivax*, the correlation between the clinical manifestations exhibited by patients with severe and non-severe complications and the genetic diversity of parasites responsible for the disease is not clear. An investigation was carried out between 2011 and 2021 on patients referred to Avicenne Hospital for suspected *P. vivax* infection. Upon arrival, they underwent clinical and biological examinations. The lateral flow test and LAMP-PCR confirmed the presence of malaria parasites, *Plasmodium* sp‥ Microscopic examination revealed the presence of *Plasmodium* parasites with a parasitaemia between 0.01 and 0.38%. Conventional PCR amplifications targeting 714 bp DNA fragment of small subunit ribosomal DNA (SSU-rDNA) followed by bidirectional sequencing allowed us to identify the parasites as *P. vivax.* The neighbor-joining (NJ) phylogenetic tree revealed that *P. vivax* sequences processed in the present study clustered in two well-differentiated and supported clades. It included a bigger clade including *P. vivax* specimens of all our patients together with homonymous sequences from Indonesia, India, and El Salvador and the second clade encompassed the sequences from Yemen and India. In addition, the clustering displayed by the median-joining network agreed well with the topology of the phylogenetic tree generated by the neighbor-joining analysis. No correlation between the clinical manifestation of patients with severe and non-severe complications, encompassing diverse geographical origins, and the genetic diversity of parasites was observed since all sequences demonstrated a high homogeneity. These findings can be helpful in getting knowledge about the population genetics of *P. vivax* and taking proper control management strategies against these parasites.

## Introduction

Malaria is a life-threatening vector-borne disease caused by protozoa of the genus *Plasmodium*, transmitted by the infected females of *Anopheles* species [1]. Up to the present, 5 species including *Plasmodium falciparum, P. vivax*, *P. ovale*, *P. malariae,* and *P. knowlesi* are known as responsible for diseases in humans [2]. Malaria is a public health issue in which nearly half of the world’s population living in 87 countries and territories are at risk [3, 4]. According to the WHO 2020 report, the number of malaria cases is estimated at about 229 millions in 2019 with mortality amounted to 409,000 deaths [3, 4]. It mostly occurs in sub-Saharan Africa followed by Southeast Asia, Eastern Mediterranean, Western Pacific, and Americas [5, 6].

*Plasmodium vivax* is the most frequent and widely distributed cause of recurring malaria. It is mainly prevalent in Central, South, and Southeast Asia, the Middle East, Latin, and South Americas, and in some restricted parts of Africa with almost 2.85 billion people at risk [7, 8]. It is commonly responsible for mild symptoms, including fever, headache, chills, or sweating [9]. These mild clinical manifestations are attributed to the fact that *P. vivax* infects only the young erythrocytes against *P. falciparum* which infects all stages of erythrocytes [10]. Nevertheless, it can cause a severe form of malaria with atypical symptoms including respiratory distress, ruptured spleen, renal failure, retinal haemorrhage, severe anaemia, thrombocytopenia, haemoglobinuria, or cerebral complications [11, 12]. *Plasmodium vivax* malaria has been overlooked over time because of its mild characteristics and lower mortality rate compared to severe *P. falciparum* malaria [13].

In order to implement effective strategies against malaria, accumulating knowledge on the genetic structure of parasites isolated from the infected individuals is essential which helps better understand the local patterns of malaria transmission and the dynamics of genetic recombination in natural *P. vivax* populations. This genetic diversity in *P. vivax* parasites can be affected by some factors such as demography of the infected populations, migration, genetic recombination, or evolutionary history of the parasite [14].

Despite several investigations carried out on the genetic diversity of *P. vivax* worldwide, most of them provided fragmentary information in the restricted areas and only four studied the genetic diversity and population structure of this parasite on a worldwide scale [15–18]. In addition, none of them argued about the probable correlation between this genetic diversity within *P. vivax* and the clinical manifestations exhibited by the patients. The aim of the present study is to determine the probable correlation between the clinical manifestations that appeared in a case series of patients infected by *P. vivax,* referred to Avicenne Hospital (Bobigny, France), and the inter*-* and intraspecific variations and the genetic diversity within *P. vivax* isolates coming from diverse geographical areas. The latter helps understand if the genetic diversity of the parasites has an impact on the severity of clinical manifestations exhibited by the patients.

## Materials and methods

### Samples and clinics

Investigation was conducted between 2011 and 2021 on the suspected patients referred to Avicenne Hospital (the northern suburb of Paris) for probable *P. vivax* infection. Upon arrival, they underwent clinical and biological examinations. Venous blood (5 mL) was collected in EDTA vacutainers from individuals with clinical symptoms reminiscent of severe and non-severe *P. vivax* malaria for diagnosis through parasitological and molecular analyses. The demographic (e.g., gender, age, location, and occupation) and clinical (medical antecedent, prescribed medication, travel history to endemic regions, and probable prophylactic measures) information were recorded for each patient individually.

### Parasitological diagnosis

May-Grünwald-Giemsa-stained thin and thick blood smears were prepared from the peripheral blood of patients and stained with 10% Giemsa for 20 min. They were examined under a light microscope (1,000× magnification) to identify malaria parasites. Parasitaemia was defined as the number of parasites detected per 10,000 red blood cells (RBCs) in a thin blood smear [19]. Microscopic examination of the isolates was further accompanied by LAMP-PCR (Alethia® Malaria, Meridian Bioscience) and lateral flow test (BinaxNOW® Malaria, Abbott, USA; VIKIA® Malaria Ag Pf/Pan, Biomérieux, France) to diagnose *Plasmodium* sp. infection [20, 21].

### Molecular characterization and typing

In order to investigate molecular characterization, genetic diversity, and population structure of the parasites isolated from patients in correlation with their exhibited clinical symptoms, the malarial parasitic DNA was extracted from peripheral blood samples using a Qiagen DNA blood kit (Qiagen, Hilden, Germany) according to the manufacturer’s protocol. It was then subjected to conventional PCR targeting small subunit ribosomal DNA (SSU-rDNA) gene, using forward (rPV1: 5’-CCGAATTCAGTCCCACGT-3’) and reverse (rPV2: 5’-GCTTCGGCTTGGAAGTCC-3’) primers with an expected length of 714 bp. Each reaction included 25 μL master mixture, containing 12.5 μL Mastermix (AmpliTaq Gold 360, Applied Biosystem), 8 μL DDW, 1 μL of each primer, and 2.5 μL of template DNA. A total of 35 cycles was performed by a PCR Thermal Cycler (Applied Biosystem, USA), under the following conditions: initial denaturation for 5 min at 95 °C, followed by 35 cycles at 94 °C for 1 min, 55 °C for 2 min, 72 °C for 90s, and final extension at 72 °C for 5 min [22]. Double-distilled water and already purified DNA isolated from *Plasmodium vivax* patients were used as negative and positive controls for each PCR batch. Amplicons were analyzed using electrophoresis in a 1.5% agarose gel containing ethidium bromide. PCR products were purified using an Invisorb Fragment CleanUp kit (Stratec Molecular, Berlin, Germany) and sequenced using the same primers for PCR amplification. The obtained sequences were edited, aligned, and blasted with GenBank database sequences to identify *Plasmodium* species. The sequences were compared to homologous sequences collected in the GenBank database and aligned with the Basic Local Alignment Search Tool (BLAST) (www.ncbi.nlm.nih.gov/BLAST). All sequences were identified based on ≥99% identity with GenBank sequences. Sequence alignment of amplified fragments using BioEdit allowed us to look for nucleotide polymorphisms. A phylogenetic analysis was carried out using MEGA v.6 software [23]. A SSU-rDNA phylogenetic tree of *Plasmodium* isolates (identified in this study) and GenBank sequences was constructed using neighbor-joining and the p-distance substitution model, supported by bootstrap values of 1,000 replicates. To display the genetic relationships within *Plasmodium* populations, the median-joining algorithm was implemented using NETWORK v. 5 software [24].

## Results

A total of 13 isolates were analyzed by clinical, parasitological, and molecular examinations. They belonged to 12 patients, one patient (AVC4) with a relapse. They were originally from Afghanistan (4 cases), Pakistan (4), France (2), Sudan (1), and India (1). The patients had an average age of 32 years old, mostly between 20 and 45 years old. Men were the most predominant patients (10 men against two women). Detailed epidemiological and clinical information of patients are given in Table 1.

**Table 1.** Epidemiological and clinical details of Plasmodium vivax-infected patients analyzed in the present study

Patient name	Epidemiological characteristics				Clinical information		
	Sex	Age (years)	Origin	Travel history	Clinical symptom	Parasitaemia	Treatment^a^
AVC1	M	33	Pakistan	Local travel	Fever	0.01	Chloroquine (10 mg/kg in d1 and d2 and 5 mg/kg in d3)/atovaquone-proguanil HCl (4*250 mg-100 mg/d for 3 days)
AVC2^b^	M	27	Afghanistan	Local travel	Abdominal pain, headache, fever, sepic shock, DIC	0.30	Artesunate (2.4 mg/kg)/chloroquine (10 mg/kg oral tablet in d1 and d2 and 5 mg/kg in d3)
AVC3	F	56	Pakistan	Local travel	Fever, sweat, abdominal pain, vomiting, headache	0.15	Chloroquine (10 mg/kg oral tablet in d1 and d2 and 5 mg/kg in d3)
AVC4^b^	M	24	Pakistan	Germany	Grippal syndrome, abdominal pain, vomiting, MAS	0.22	Quinine (8 mg/kg)/piperaquine-artenimol (4*320 mg-40 mg/day for 3 days)
AVC5	M	20	Sudan	Local travel	Abdominal pain, fever, headache, dark urine	0.12	Chloroquine (10 mg/kg oral tablet in d1 and d2 and 5 mg/kg in d3)
AVC6^b^	M	25 (AVC4 relapse)	Pakistan	Local travel	Abdominal pain, headache, fever, septic shock	0.38	Quinine (8 mg/kg)/piperaquine-artenimol (4*320 mg-40 mg/day for 3 days)
AVC7	F	45	France	Pakistan	Headache, sweat	0.05	Chloroquine (10 mg/kg oral tablet in d1 and d2 and 5 mg/kg in d3)
AVC8	M	34	Afghanistan	Iran, Turkey, Greece, Italy	Fever, headache, abdominal pain, vomiting	0.16	Piperaquine-artenimol (4*320 mg-40 mg/day for 3 days)
AVC9	M	24	Afghanistan	Local travel	Fever, headache, asthenia, confusion (chloroquine intolerence)	0.30	Quinine (8 mg/kg)/piperaquine-artenimol (4*320 mg-40 mg/day for 3 days)
AVC10	M	23	India	Local travel	Fever, sweat, headeache	0.13	Piperaquine-artenimol (4*320 mg-40 mg/day for 3 days)
AVC11	M	27	Pakistan	Iran, Turkey, Balkan	Fever, diffuse pain, conjunctival icterus, headache	0.06	Chloroquine (10 mg/kg oral tablet in d1 and d2 and 5 mg/kg in d3)
AVC12	M	67	France	Iraq, Yemen	Asthenia, headache, fever, diffuse pain	0.30	Piperaquine-artenimol (4*320 mg-40 mg/day for 3 days)
AVC13	M	8	Afghanistan	Local travel	Vomiting, fever, sweat	0.05	Artemether-lumefantrine- (3*20 mg-120 mg/12h for 2.5 days)

Abbreviations: DIC, diffuse intravascular coagulation; MAS, macrophage activation syndrome.

a All processed patients were treated with primaquine (30 mg/d for adults and 0.5 mg/kg/d for infants for 14 days) as a complementary treatment to avoid relapse.

b **Patients with severe *P. vivax* malaria.**

Based on the clinical examinations, three patients (AVC2, AVC4, and AVC6) exhibited clinical symptoms resembling severe malaria while nine patients possessed non-severe malaria according to the WHO recommendation [3, 25]. Diffuse intravascular coagulation (DIC), macrophage activation syndrome (MAS), and septic shock were such of the symptoms observed in patients with severe malaria. All patients had parasitaemia inferior to 0.4%. No correlation was observed between the level of parasitaemia and the severity of clinical pictures, but the severe cases were among patients with highest parasitaemia (0.3 to 0.38%). Clinical symptoms observed in patients with severe versus non-severe *P. vivax* malaria are given in Supplementary Table SI-1.

Microscopic examinations revealed an infection in patients with *P. vivax.* Morphological identification was carried out based on some criteria such as enlarged infected erythrocytes and the appearance of granules, called ’Schüffner’s dots’, over the erythrocyte cytoplasm. Parasitological analyses by LAMP-PCR and lateral flow test further confirmed the infection by *Plasmodium* sp. parasites.

In order to confirm the identity of parasites and determine inter- and intraspecific genotypic relationships between our isolates and those reported from other endemic regions, the *Plasmodium* isolates were subjected to conventional PCR targeting SSU-rDNA. All isolates found positive after microscopic examination were also positive by PCR. Bidirectional sequencing allowed the identification of parasites at the species level as *P. vivax.* All sequences were deposited in GenBank under the assigned accession numbers from XK542981 to XK542994.

**Figure 1.**
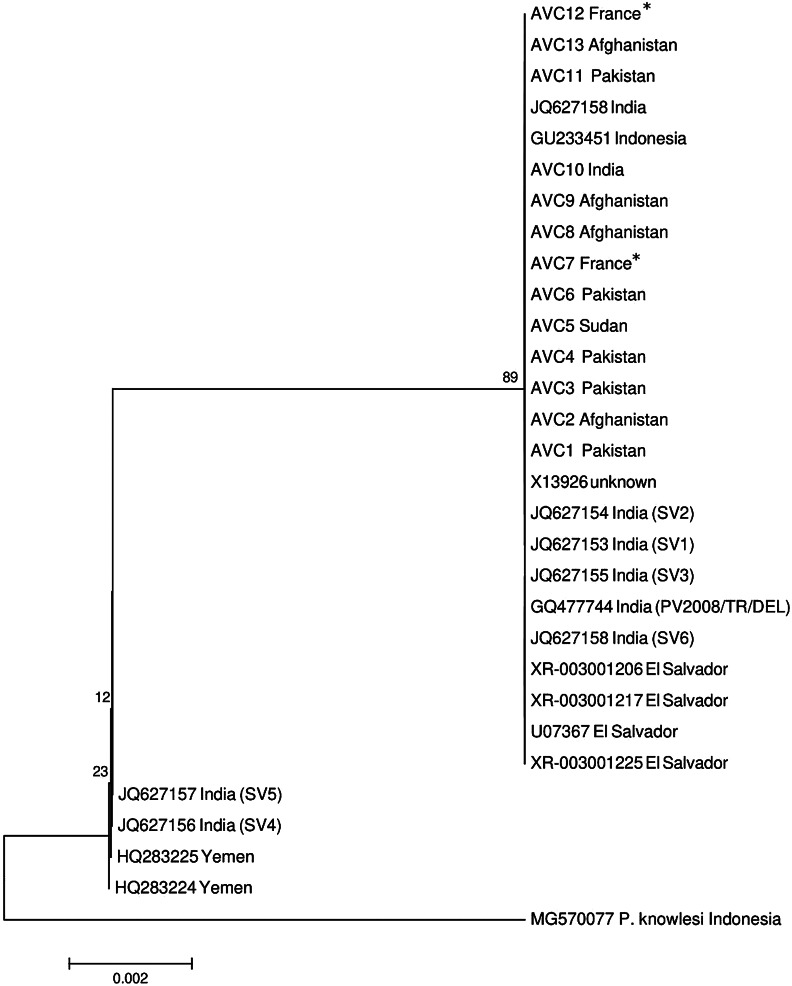
Neighbor-joining (NJ) phylogenetic tree constructed by SSU-rDNA sequences of P. vivax specimens analyzed in this study (samples entitled AVC) together with those from GenBank. *: Two French patients with a history of recent travel to Pakistan, Iraq, and Yemen.

**Figure 2.**
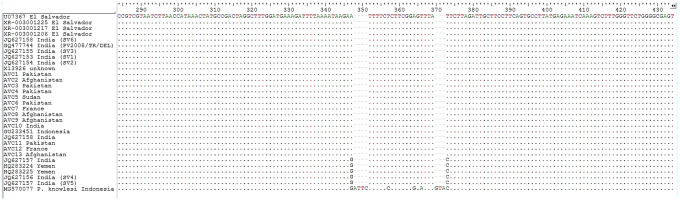
Single nucleotide polymorphism (SNP) observed in P. vivax sequence alignment of our patients and GenBank sequences.

**Figure 3.**
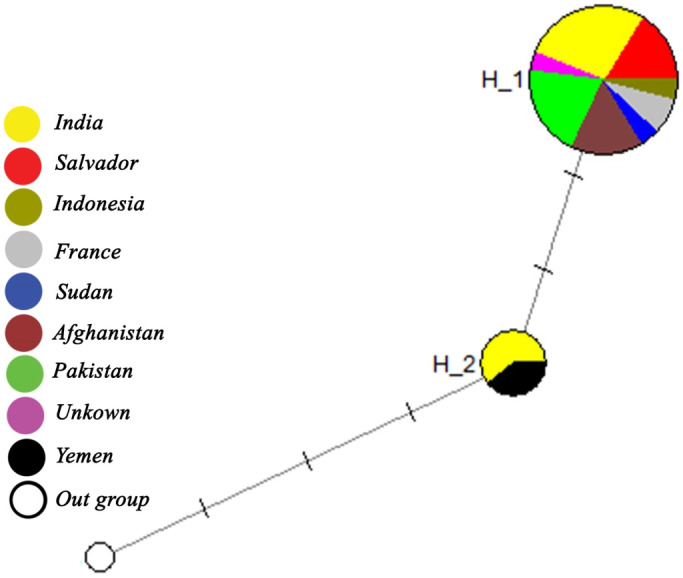
Median-joining network deduced from P. vivax SSU-rDNA sequences. Circle size and color are indicative of the frequency and the geographical location of haplotypes. Haplotype identification is provided next to the corresponding circle.

Based on the NJ phylogenetic tree generated from our sequences and those from GenBank, the *P. vivax* isolates were clustered in two well-differentiated and supported clades (Figure 1). The first bigger clade included *P. vivax* specimens of all our patients together with homonymous sequences from Indonesia (GU233451), India (JQ627153-JQ627155, JQ627158, and GQ477744), and El Salvador (XR-003001206, XR-003001217, XR-003001225, and U07367). The second clade encompasses the sequences from Yemen (HQ283224 and HQ283225) and India (JQ627157, JQ627156, and JQ627157). The sequence alignment of the isolates revealed the presence of two SNPs (single nucleotide polymorphisms) which explains the presence of two subpopulations of *P. vivax* in some countries such as India (Figure 2). In addition, the clustering displayed by the median-joining network was in accordance with the topology of the phylogenetic tree generated by the neighbor-joining analysis (Figure 3). The distribution of SSU-rDNA haplotypes within *P. vivax* sequences processed in the present study is shown in Table 2.

**Table 2.** Distribution of SSU-rDNA haplotypes within the *P. vivax* populations analyzed in this study

Haplotype	Numbers	Country	Total
H1	25	Salvador	4
		India	9
		Unknown	1
		Pakistan	5
		Afghanistan	4
		Indonesia	1
		France	1
H2	5	India	3
		Yemen	2
Total			30

## Discussions

France has one of the highest numbers of malaria cases reported in returned travellers, with about 5,000 cases per year [26, 27]. Around 95% of malaria cases are observed in people returning from malaria-endemic countries [27]. Patients with *P. vivax* make up 4% of the total number of imported cases [12]. In the present study, 10 of 12 patients were immigrants from endemic countries mostly from Afghanistan and Pakistan. Two cases were French traveller patients with a history of recent travel to Pakistan, Iraq, and Yemen. Furthermore, most of the processed individuals were men which points to the fact that the majority of immigrants are men [28] (Table 1).

*P. vivax* malaria is known to possess mild complications [10]. With less than one severe case per year on average, *P. vivax* is very rarely associated with severe imported malaria in France [12]. Dramatically, 3 of 12 patients exhibited clinical symptoms such as macrophage activation syndrome (MAS), diffuse intravascular coagulation (DIC), and septic shock based on RPC 2017 and WHO 2020 [3, 25] recommendations, with the latter two implying to severe malaria. Although some other complications like impaired consciousness, respiratory distress, multiple convulsions, prostration, pulmonary oedema, abnormal bleeding, or jaundice have been reported for *P. vivax* severe malaria [29], they were absent in our patients. Therefore, contrary to the benign reputation of *P. vivax* malaria, its clinical manifestation is not always very mild, inciting acute infection with septic shock, MAS, or DIC. Furthermore, one of the patients of Pakistani origin exhibited a relapse one year after the first infection (Table 1). Except for the above-mentioned patient, no case of multiple infections from the same individual was noticed in this study. In endemic areas, relapse of *P. vivax* malaria is a major cause of malaria in young children and is an important source of malaria transmission which can appear even more than 5 years after initial contamination [30, 31]. Furthermore, most of the patients had a history of local travel to countries of their homeland.

The malaria epidemiology is influenced by environmental factors (e.g., temperature and rainfall) and socioeconomic conditions. Besides, other factors such as urbanization, exponential population growth, instability, military conflicts, migration, and environmental changes due to excessive rains or floods, and extensive irrigation projects favour malarial parasite transmission as well [32]. Afghanistan and Pakistan are the endemic foci with a high burden of malaria. The eco-geographical diversity in Afghanistan contributes to the heterogeneous prevalence of malaria across the country. Approximately 60% of the population (nearly 14 million people) lives in malaria-endemic areas [33]. Eighty-five percent of the whole malaria cases are prevalent in 63 of 400 districts. Most of them (Nangarhar, Kunar, Nuristan, Khost, Paktika, and Laghman) are located along the border with Pakistan [34]. *P. vivax* is a prominent species in Afghanistan causing more than 95% of all malaria cases [35]. Military conflicts and instability together with living at unsuitable locations, lack of means for personal protection, and difficulty of access to healthcare are such elements which favour the emergence of vector breeding sites, population movements, and a high burden of malaria. In Pakistan, malaria is one of the most devastating parasitic diseases with 110 million individuals at risk and an estimated incidence of 500,000 cases and 50,000 deaths annually. *P. vivax* is the most prevalent species (88%) followed by *P. falciparum* (12%) [36]. According to the latest stratification, 66 districts have been categorized in the high endemicity stratum (annual parasite incidence >5 per 1,000) in which those located in the northern part of the country (e.g., Federally Administered Tribal Areas (FATA) and Balochistan and Khyber Pakhtunkhwa (KP) provinces) possess the highest burden of malaria [36–38]. Many factors have contributed to an increase in malaria cases in these regions including warm autumns (resulting in extended transmission period), the emergence of chloroquine resistance across the country, and a chronic decline in vector control activities [39, 40]. In addition, the migration of people from malarial endemic regions to less or non-immune communities can lead to the serious threat of malaria reintroduction in malaria-free areas [41, 42]. Five of eight patients from Afghanistan and Pakistan in this study come from this buffer endemic zone on the Afghan-Pakistani border.

Despite the wide distribution of *P. vivax* as the most frequent species worldwide, a comprehensive picture of the global genetic diversity and population structure of *P. vivax* has been poorly studied. Although several local investigations have been conducted on the genetic diversity of *P. vivax*, they provide only a piece of fragmentary information without giving a global image. In this descriptive analytic study, we aim at evaluating the correlation between the genetic diversity of *P. vivax* and the clinical symptoms of patients with severe and non-severe infections. We therefore did not evaluate the factors associated with the pathogenicity or severity of causative *P. vivax* by molecular analysis. Despite the relatively low specimen numbers examined, we found a genetic diversity among our isolates compared to those from other endemic countries. In analyzing the NJ phylogenic tree generated with specimens of our patients together with GenBank sequences, we recorded a genetic heterogeneity among the processed sequences leading to a cluster with their counterparts in two clades (Figure 2). These findings are consistent with the results of other studies indicating the presence of two lineages categorized as Old World and New World, based on geographical subdivision and genetic and phenotypical markers. These lineages are not confined geographically and are present worldwide [43]. In another investigation on *P. vivax* patients in Southern Thailand, a high level of genetic diversity within *P. vivax* specimens was also reported using three antigenic markers and eight microsatellite markers [44]. Afghanistan and Pakistan isolates were demonstrated to have a high homogeneity while a degree of genetic separation was observed for some isolates from India (Table 2). This finding supports the results of Benavente et al. [18], in which Afghanistan and Pakistan isolates were clustered together in the same clade. Despite the geographically close distance, the highest genetic diversity in *P. vivax* isolates was observed in sequences from India which led to grouping of Indian samples in two subpopulations. A genetic separation was observed in some Indian isolates compared to Pakistan and Afghanistan specimens [18]. In the study conducted by Rougeron et al. [16], Pakistan’s isolates were positioned in a clade far from Indian isolates. Nevertheless, this genetic separation was valid for some of our samples but not for all. Consequently, the correlation between the genetic diversity and geographic distance from Central Asia (India) remained highly significant [17]. On the other hand, no correlation between the clinical manifestation of patients with severe and non-severe complications and the genetic diversity of parasites was observed since all sequences demonstrated a high homogeneity (Figure 2). In sequence alignment of isolates processed in this study with those coming from GenBank, two SNPs were observed which explain the presence of two subpopulations of *P. vivax* in some countries such as India (Figure 2). These findings were further supported by network analysis (Figure 3).

Patients processed in the present study were undergoing treatment with chloroquine (10 mg/kg oral tablet in d1 and d2, and 5 mg/kg in d3) for non-severe *P. vivax* malaria and quinine (25 mg/kg/d) and artesunate (2.4 mg/kg) for severe cases, and a favourable outcome was observed within 7 days post-treatment (Table 1).

## Conclusion

Although *P. vivax* is known as a pathogen with mild clinical manifestations, we provide further evidence on the exhibition of severe complications (diffuse intravascular coagulation (DIC), macrophage activation syndrome (MAS) and septic shock) in 3 of 12 studied patients. In addition, we highlight heterogeneity within isolated parasites using NJ phylogenetic analysis in which *P. vivax* sequences clustered in two well-differentiated and supported clades. The first clade includes *P. vivax* specimens of all our patients together with homonymous sequences from India, Indonesia, and El Salvador, and the second clade encompasses sequences from Yemen and India. Furthermore, no correlation between the clinical manifestations of patients with severe and non-severe complications and the genetic diversity of parasites was observed. However, these findings are limited to the restricted number of patients analyzed in this study. These results can be supported by a larger scale sampling in terms of geographical locations from France and possibly other endemic countries, by evaluating other demographic factors (e.g., sex and age) and by looking for other molecular markers, particularly in relation with the pathogenicity of these parasites.

## Supporting information

Soumaoro et al. supplementary material Soumaoro et al. supplementary material

## Supplementary material

For supplementary material accompanying this paper visit http://dx.doi.org/10.1017/S0950268823001632. click here to view supplementary material
